# A Statement in Regard to Several Puerperal Fever Cases, as Read before the Indinapolis Medical Society, June 1st, 1850

**Published:** 1851-07

**Authors:** T. Bullard


ARTICLE II.

### *A Statement in regard to several Puerperal Fever Cases, as read before the Indianapolis Medical Society, June 1st, 1850.*—By T. Bullard, M.D.

With your permission, I will make a few brief statements, in regard to a matter of much interest to myself, and which may be the occasion of eliciting the expression of opinions and facts of value to the Society.

From the 25th of March to April 14th, I attended five cases of Obstetrics—but one of them in town, and this manifested a slight febrile tendency, which, however, required but two or three days attendance. Those in the country, did not demand a second visit, save one, and that for an attack of tonsilitis within the mouth. From April 14th to May 23d, I attended nineteen cases, ten of which were within the corporation. Of the nineteen, ten had puerperal fever. Of these I have had five fatal cases, and I have learned that one other died whom I delivered, but which fell into the hands of another physician, on the third day after delivery, while I was ill.

My first fatal case, was a woman of bad constitution—scrofulous—attacked on the 3rd day and died on the 12th; sinking from secondary causes—prostration, etc., from uncontrolable irritation of the bowels.

Second case attacked on the 3rd day and died on the 6th. Rapid peritonitis, great effusion. 3rd attacked on the 2d day and died on the 5th; 4th attacked on the 2d day and died on the 4th; 5th attacked on the 5th and died on the 6th; showing that the sooner febrile symptoms showed themselves after delivery, the more rapidly fatal were the cases.

The most severe case I have had which recovered, was not attacked until the 5th day, and on the 6th day the peritoneal inflammation seemed to be transferred to the pleura. Active depletion, etc., controled this, and in a few days she was convalescent.

The 3d and 4th cases had great gastric irritation and regurgitation of every thing taken into the stomach, with a dark green fluid, much such as I have seen in the black vomit of the yellow fever.

Now, without detaining you, there are a few questions of interest arising from the foregoing facts. For nearly six years, during which time I have been in practice in Indiana, I have had a reasonable share of obstetric practice; and until last month, have not lost a single patient from Puerperal Fever, and but two, I believe, in child-bed—one in the last stages of the Consumption, and the other from congestion in Autumnal fever. Why then this sudden, and to me somewhat trying loss of *five*, within as many weeks? Unless desired, I will not enter upon the treatment, which, to the best of my judgment, was in accordance with the received principles of practice, which, by-the-way, I admit is not saying much, for one can find authority for any mode of practice, and one about as successful, or rather *un*-successful as another. In three of the fatal cases, I had counsel, and offered it in all save one, and in that it was so obviously useless, I did not wish to involve any one else in the responsibility.

First, then, is it epidemic? and if so, is it confined to the immediate vicinity? I believe numbers present, will be able to aid in the answer of this query.

Second, if not epidemic, is it contagious, communicable, *portable* or transmissible by the physician, nurses or friends, passing from one puerperal woman to another?

I have had perplexing doubts on this subject, and there are some weighty reasons for and against. I do not here allude to the testimony of authors on the subject, although I have carefully examined them, for I am not writing an essay upon this disease. I have not a thing to hide in this matter, and wish the truth to be known.

If no other physician in town has had marked cases of this disease since April 14th, or about that time, except those who may have been in connection with patients whom I have attended since then, it would seem strongly to show that the "fons et origo," as the lamented Harrison would say, was the first fatal case I had, and that all others had in some way been derived from that.

In favor of this view, it should be stated, that in the family of my first fatal case, at the time of her confinement, her little daughter, of six or seven years of age, was ill of an ordinary intermittent, and about the third day after febrile symptoms manifested themselves in the mother; severe *erysipelas* took the place of the intermittent, which proved a malignant and obstinate case, yet which ultimately recovered—looking not only towards its communicability, but satisfying the opinion that the virus of the one disease may produce the other.

On the other hand, why have not my country patients been attacked, if it is transmissible, and not locally epidemic? True, I understand that two of these were so attacked, of whom one died and one recovered, and that the physician who attended them while I was ill, has since had at least one fatal case in his own practice with which I had no intercourse until a few hours before death. I shall be glad to hear anything which shall shed light upon this obscure, and to me, interesting matter. I shall also be most happy freely to answer, as far as I am able, any inquiries in relation to my cases, and beg to be permitted to express my thanks to those of my brothers in the profession who have so freely and unsought, given me their countenance and sympathy in this dark period of my professional carreer among you. You all well know, that it is the dark hour,—the one of trial and misfortune, if you will, which ties one's friends, and I am happy to say that mine have not been only fair-weather friends.

I may add, that the character of the labor, either in regard to time or severity, seeemed to have no influence whatever, in inducing an attack. Indeed, the worst case I had, was delivered a few minutes before my arrival, while in the act of relieving her bowels, not admitting she was in labor, so that I had nothing to do but put her to bed and deliver the placenta from the vagina by gentle traction upon the chord. Nor was ergot used in but one case attacked, while it was used in two or three not attacked at all.

Fearing, as the disease seemed to trace me alone, that possibly I might communicate the poison, and being far from well, I determined to absent myself from town. I consequently left for Peru, Logansport, Lafayette, etc., and was gone some nine days. On the next day after my return, with every article of apparel *new*, I ventured to attend an obstetrical case, believing most sincerely, that so far as contagiousness was concerned, I ran no risk of communicating the disease. Notwithstanding, she was seized with puerperal fever and died on the 5th day—and the same result in regard to an Irish woman whom I delivered on the 3d day after my return ! I now refused to attend all obstetric cases, till about the first of September. Since then I have been gradually resuming my obstetric practice, and thus far satisfactorily.

Meanwhile, I believe our country has not been entirely free from a tendency to puerperal fever and erysipelas, which last especially might be regarded as epidemic during the last of winter and early spring. Other of our physicians I believe, have had a number of puerperal cases, occasionally, ever since last spring; and it is well known that an unusual number of women have been lost within the last twelve months, in child-bed. I have seen several cases since last June occurring in the practice of other physicians, some of which died and others recovered. My opinion is, that the disease is now on the decline, and when it does occur, it is milder and more answerable to treatment. Still, our best physicians, occasionally have a fatal case.

If hereafter, the above brief record of facts should afford any relief to a brother practitioner in the way of precedent, showing that he is not the first, thus, almost alone to encounter the onset of such a malady, I shall be more than repaid.—Such relief, I certainly did obtain from the perusal of Meig's article on Puerperal Fever, especially form Dr. Rutter's experience, to which I beg leave to refer.

